# Distinct Behaviour of the Homeodomain Derived Cell Penetrating Peptide Penetratin in Interaction with Different Phospholipids

**DOI:** 10.1371/journal.pone.0015819

**Published:** 2010-12-30

**Authors:** Ofelia Maniti, Isabel Alves, Germain Trugnan, Jesus Ayala-Sanmartin

**Affiliations:** 1 CNRS, UMR 7203, Laboratoire des Biomolécules, Groupe N. J. Conté, Paris, France; 2 Université Pierre et Marie Curie Paris 6, Paris, France; 3 École Normale Supérieure, Département de Chimie, Paris, France; University of South Florida College of Medicine, United States of America

## Abstract

**Background:**

Penetratin is a protein transduction domain derived from the homeoprotein Antennapedia. Thereby it is currently used as a cell penetrating peptide to introduce diverse molecules into eukaryotic cells, and it could also be involved in the cellular export of transcription factors. Moreover, it has been shown that it is able to act as an antimicrobial agent. The mechanisms involved in all these processes are quite controversial.

**Methodology/Principal Findings:**

In this article, we report spectroscopic, calorimetric and biochemical data on the penetratin interaction with three different phospholipids: phosphatidylcholine (PC) and phosphatidylethanolamine (PE) to mimic respectively the outer and the inner leaflets of the eukaryotic plasma membrane and phosphatidylglycerol (PG) to mimic the bacterial membrane. We demonstrate that with PC, penetratin is able to form vesicle aggregates with no major change in membrane fluidity and presents no well defined secondary structure organization. With PE, penetratin aggregates vesicles, increases membrane rigidity and acquires an α-helical structure. With PG membranes, penetratin does not aggregate vesicles but decreases membrane fluidity and acquires a structure with both α-helical and β–sheet contributions.

**Conclusions/Significance:**

These data from membrane models suggest that the different penetratin actions in eukaryotic cells (membrane translocation during export and import) and on prokaryotes may result from different peptide and lipid structural arrangements. The data suggest that, for eukaryotic cell penetration, penetratin does not acquire classical secondary structure but requires a different conformation compared to that in solution.

## Introduction

Cell penetrating peptides (CPP) and Protein Transduction Domains (PTDs) are potential therapeutic vectors for the delivery of molecules inside eukaryotic cells (for review see [1]–[3]). These peptides are alternative to more “aggressive” methods used to introduce molecules into cells such as trituration [4] and microinjection. Such peptides (i.e. Tat, penetratin, polyarginine) are usually rich in basic amino acid residues, and some of them are derived from proteins suggesting that they play a role in messenger protein transduction [5]. Penetratin, a peptide derived from the homeodomain transcription factor Antennapaedia was described as one of the first peptides to successfully carry active molecules inside cells and is one of the most studied PTDs [6]–[8].

Different physicochemical parameters are involved in membrane binding and penetration of CPPs [9]. Cell penetration is known to be independent from receptors and metabolic energy. Several studies have demonstrated that endocytosis is also involved in the internalization of basic peptides [10], [11]. However, to reach the cytosol and the nucleus, the peptides must escape from the endosome through the endosomal membrane barrier. Thus, a direct interaction with membrane lipids seems to be important for their cytosolic or nuclear localization.

Several mechanisms for CPP membrane translocation have been proposed. These include an “electroporation-like” mechanism [12], neutralization of arginine residues by guanidinium-phosphate complex formation [13], and inverted micelles formation [14] (for reviews see [1], [2], [15]). However, the electroporation mechanism has been contested and recently a direct translocation through the bilayer has been suggested [16]. Experiments with model membranes have established that the translocation in large unilamellar vesicles (LUVs) is dependent on membrane potential and is modulated by the lipid composition [17]. However, in giant unilamellar vesicles (GUVs), membrane translocation was not dependent on membrane potential [18], [19]. This difference of potential sensitivity may be related to membrane curvature and/or membrane tension that are higher in LUVs than in GUVs. A more positively curved membrane will need a driving potential that may not be necessary for a flat membrane. Using membrane models, we have previously shown that penetratin and different basic peptides induce membrane invaginations which results in the formation of tubular structures [20]–[22]. We suggested that membrane curvature induced by basic peptides could be crucial to their mechanisms of internalization [23]. Positive curvature-induction would be necessary for pore formation of amphipathic peptides. Negative curvature would be related to the formation of tubes (“physical endocytosis”) [20] and inverted micelles. Another important property of basic peptides is their capacity to aggregate membranes. This property observed for several peptides [20], [24] shows that a peptide can be covered by phospholipids and therefore could be related to the peptide induced formation of very thin tubes and inverted micelles.

With regards to the protein transduction domains present in transcription factors (i.e. penetratin), it should be considered that these molecules may be able to cross the plasma membrane for their internalization and also for their release to the extracellular medium by the cells. Therefore, the basic domain has to be able to interact with the external leaflet of the plasma membrane rich in phosphatidylcholine (PC) for cell import, and also the internal leaflet rich in phosphatidylethanolamine (PE) and negatively charged phospholipids such as phosphatidylserine (PS) for cellular export. Notice that the external leaflet of the eukaryotic cells has (even if it is in low abundance), negatively charged lipids. Moreover, the recently observed antimicrobial activity of penetratin suggested its interaction with PE and phosphatidylglycerol (PG) rich membranes [25].

Besides the capacity of peptides to modify the arrangements of membrane phospholipids, peptide structural changes might be important for membrane translocation. For example Pep-1 and pVec adopt an α-helix when associated to phospholipids [26], [27]. Penetratin has been extensively studied by a circular dichroism (CD) approach. The results show that penetratin is able to acquire α–helix and β–sheet conformations in different conditions [24], [28]–[33].

In this study, penetratin actions on phospholipids typical of the extracellular leaflet of eukaryotic cells (PC), of the intracellular leaflet (PE) and of the microbial membranes PG and PE) were investigated. We analyzed the penetratin capacity to modify the membrane lipid organization by Fluorescence and Infrared spectroscopy, by plasmon waveguide resonance and by differential scanning calorimetry. The accompanying peptide structural changes were studied by Circular dichroism and Fourier transformed Infrared spectroscopy. The results indicate that penetratin is able to induce different peptide-lipid arrangements depending on the type of phospholipid. The implications of the presented data in penetratin membrane activities are discussed.

## Results

### Penetratin-induced vesicle aggregation

**Figure 1:**
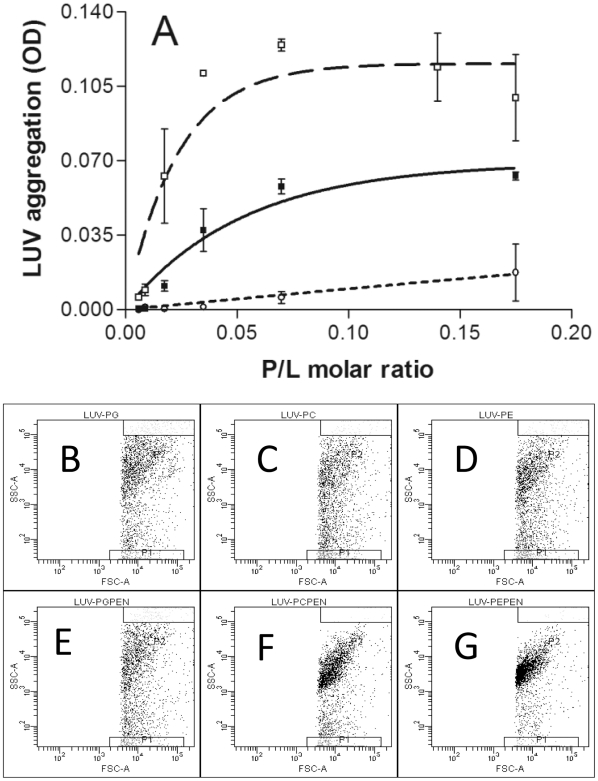
Vesicle aggregation by penetratin. A) LUVs aggregation was measured by turbidimetry at plateau as a function of peptide/lipid molar ratio. PE (□); PC (▪); PG (○). Flow cytometry analysis of PG, PC and PE LUVs populations. Notice that the size and granulocity are equal for the three LUVs (B,C,D). After penetratin addition PG LUVs distribution does not change (E), but the dot plot distribution for PC (F) and PE (G) change due to LUVs aggregation. (Representative of 3 and 2 experiments).

To quantify penetratin ability to provoke membrane bridging, we measured the aggregation of PC, PE and PG LUVs by monitoring the turbidity of the sample (Fig. 1A). Penetratin induced a strong and progressive increase in the turbidity of the PE suspension starting from a rather low peptide/lipid molar ratio (1/100). At a peptide/lipid ratio of 1/30 the OD reached a plateau. An important increase in OD was also observed following penetratin addition to PC LUVs, starting at a peptide/lipid molar ratio of 1/50. The plateau was reached at a peptide/lipid ratio of 1/15. At the lipid concentrations used in these experiments (20 µg/ml lipids), penetratin induced only a marginal increase in the absorbance of a PG LUVs suspension. Penetratin-induced aggregation of PG vesicles was only observed for lipid concentrations higher than 0.5 mg/ml (at peptide/lipid ratios higher than 1/7 not shown).

LUVs aggregation was also studied by flow cytometry. As shown in figure 1, the dot plots of PC, PG and PE LUVs were quite similar indicating that the size and granulocity of these LUVs were similar (Fig. 1B,C,D). The addition of penetratin to the LUVs suspensions changed the dot distribution of PE and PC LUVs. The vesicle population was strongly concentrated for PE and a smaller but evident effect was also observed for PC LUVs (Fig. 1F,G). On the contrary, PG LUVs showed no significant change in dot distribution after penetratin addition indicating the absence aggregation (Fig. 1E).

### Penetratin interaction with planar lipid bilayers

Plasmon waveguide resonance (PWR) permitted us to obtain information about peptide binding affinity and to follow the peptide-induced changes in the lipid mass density and organization. From the spectral changes (using the resonance minimum position), upon incremental addition of peptide, an apparent dissociation constant for the interaction of the peptide with the membrane was obtained. Apparent, because upon peptide binding to the membrane, two processes occur: mass and structural changes of the peptide itself and mass and structural changes of the lipid bilayer to accommodate the peptide. A second type of information can be obtained with this technique that arises from the use of both perpendicular *p*- and parallel *s*-polarized light to create resonances, which allows characterization of the mass and structural changes induced by the peptide on the membrane.

Numerical values of the PWR spectral changes occurring after addition of penetratin to the membrane bilayers of different composition are shown in Table 1. As previously reported [34], the binding of penetratin to the PC bilayer produced a biphasic event, with a decrease in the resonance angle position both for *p*- and *s*-polarization at low concentrations (up to 0.1 µM), followed by positive shifts for both polarizations at higher concentrations. From the second binding event a binding affinity has been calculated, with a Kd of 0.6 µM. The low concentration event was characterized by a large decrease in the resonance minimum that after graphical analysis has been mainly attributed to a decrease in mass which can only be explained by an efflux of lipids into the plateau Gibbs border [35]. In the second binding event, positive shifts were observed for both polarizations, mainly related to mass changes (80%, increase) and some structural changes (20%) (Table 1). We propose that those correspond to a rearrangement of the peptide and the lipids with lipid influx into the bilayer core and repacking of the lipids around the peptide.

**Table 1:** Effects of penetratin on PC, PC/DOPE (1/1) and PG bilayers observed by PWR.

Lipid	PC		PC/DOPE		PG
Binding process	1st	2nd	1st	2nd	only one
Spectral change in *p* (mdeg)	−19	+24	−12	+15	+34
Spectral change in *s* (mdeg)	−25	+32	−18	+17	+38
Mass-related change	76%	80%	76%	84%	85%
Structural-related change	24%	20%	24%	16%	15%
Affinity (Kd)		0.6 µM		0.01 µM	0.04 µM

The interaction of penetratin with the zwitterionic PC/DOPE bilayer produced, like for PC, two binding events with negative shifts followed by positive shifts for both polarizations (Table 1). A Kd of 0.01 µM was obtained, indicative of a high affinity of penetratin for this lipid composition. The magnitudes of the spectral changes of both events were smaller than those observed in the case of PC. Concerning the first binding event, this could be related with the fact that PE has a smaller head group than PC and so induces smaller lipid rearrangements. The two binding events were associated with a large mass change and a structural change component.

In the case of PG, only one binding event was observed, penetratin led to positive shifts for both *p*- and *s*-polarizations (Table 1). A considerable enhance in the binding affinity was observed, when compared with the zwitterionic PC, with a Kd of 0.04 µM. This binding event is characterized mainly by a change (increase) in mass that cannot be solely explained from the peptide weight itself (as it could not lead to such large spectral change, considering its small mass) but could arise from an efflux of the lipid from the plateau Gibbs border into the membrane. With PG the first binding event observed with PC and PC/DOPE was absent because there are no repulsive interactions in this case between the peptide and the lipid head groups but rather attractive ones are established between the positively charged amino acids and negatively charged lipid. The magnitude of the spectral changes (Table 1) are slightly larger than those observed in the second binding event of penetratin to PC and result from a higher reorganization of the lipid (higher packing) around the peptide due to favourable electrostatic interactions.

The differences in peptide ability to produce membrane aggregation did not seem to correlate with a difference in the peptide-membrane affinity but correlated with the binding process; biphasic for PC and PC/DOPE and monophasic for PG (no aggregation). These facts suggested that different mechanisms according to lipid polar head charge and structure were involved. Therefore, we investigated peptide-induced changes on membrane organization and whether these changes were related to differences in peptide structure.

### Membrane fluidity alterations after penetratin binding

To evidence possible modifications in membrane fluidity induced by penetratin interaction with lipids we used the fluorescent probe Laurdan. When inserted in membranes, Laurdan distributes equally between lipid phases and displays a phase-dependent emission spectral shift, from 440 nm in the ordered lipid phase to 490 nm in the disordered lipid phase [36]–[38]. This effect is attributed to reorientation of water molecules present at the lipid interface near Laurdan's fluorescent moiety, i.e., water dipolar relaxation process.

**Figure 2:**
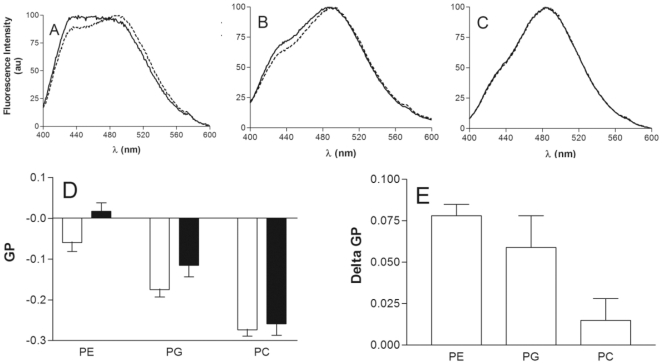
Modifications in liposome-Laurdan fluorescence induced by penetratin. Normalized fluorescence spectra of Laurdan in PE (A), PG (B) and PC (C) LUVs in the absence (dotted line) or presence (continuous line) of penetratin at 1/25 P/L molar ratio. D) Calculated GP values for PE, PG and PC LUVs in the absence (white) or presence of penetratin (black). E) Delta GP induced by penetratin on PE, PG and PC LUVs. (Mean of 3 experiments).

In our case, Laurdan emission spectra in PC, PE or PG vesicles presented two fluorescence maxima (Fig. 2) at 430 nm and 490 nm, attributed to the fluorescence emission of Laurdan molecules in the ordered and fluid phase, respectively. The presence of penetratin induced an increase in the fluorescence emission intensity at 430 nm on PE (Fig. 2A) and PG (Fig. 2B) LUVs. With PE, penetratin induced also a relative decrease in the intensity at 490 nm. No significant changes were observed for PC LUVs (Fig. 2C).

The GP parameter permitted us to quantify the effect of the peptide (Fig. 2D,E). In the case of PE LUVs the GP increased from −0.060 in the absence of peptide to 0.017 in its presence (ΔGP = 0.077±0.007). For PG LUVs, GP also significantly increased from −0.175 to −0.116 in the presence of peptide (ΔGP = 0.059±0.019). Thus, penetratin had an ordering effect on both PE and PG LUVs. This effect was stronger for PE and non significant for PC LUVs (ΔGP = 0.019±0.013). The presented experiments were performed at 37°C but similar results were obtained at 25°C (not shown).

### Peptide effect on lipid phase thermal transition

The interaction of the peptide with lipids was also monitored by following the changes in lipid phase pre-transition arising from the conversion of L_β_' to P_β_', and the main phase transition corresponding to the conversion from P_β_' to Lα (T_m_) upon peptide/lipid interaction. Most molecules that interact with lipids affect the pre-transition that arises from an alteration in the head group tilting. As for the main transition, its enthalpy is mainly due to the disruption of van der Waals interactions between the fatty acid chains, and perturbations on this transition are indicative of intercalation of the peptide between the fatty acid chains. Molecules that perturb the main phase transition often decrease the cooperativity of the phase transition characterized by the transition half width. The studies presented here were performed with P/L molar ratios of 1/100, 1/50, 1/25 and 1/10.

The DMPC pre-transition was abolished in the presence of penetratin at P/L ratio of 1/10 (not shown) and the cooperativity of the main transition was affected from P/L 1/25. Tm increased from 23.4°C for the lipid alone to 24.7°C in the presence of peptide at the highest P/L ratio, reflecting a small rigidification of the membrane by the peptide (Table 2). Overall, the perturbation by the peptide of the phase transition was small, indicating a rather superficial interaction of the peptide in the lipid surface without penetration in the fatty acid chain region.

**Table 2:** Thermodynamic parameters for the interaction of penetratin with MLVs of different composition and at different P/L ratio.

	Lipid Alone		P/L (1/100)^a^		P/L (1/50) a		P/L (1/25) a		P/L (1/10) a	
	Tm (°C)	ΔH (kcal/mol)	Tm (°C)	ΔH (kcal/mol)	Tm (°C)	ΔH (kcal/mol)	Tm (°C)	ΔH (kcal/mol)	Tm (°C)	ΔH (kcal/mol)
DMPC	23.4	6.6	23.1	7.6	23.2	7.5	24.0	7.4	24.7	6.1
DMPG	32.7	5.6	36.5	4.9	37.8	3.3	38.5	3.7	41.3	0.4
DMPE	49.5	5.8	51.6	5.5	52.1	4.5	52.9	3.8	53.3	3.0

*a*; Here MLVs were used instead of LUVs and the peptide interacts only with the most external lipid layer. Therefore, the P/L ratios indicated here are overestimated.

The DMPG thermogram (in the absence of peptide) was not symmetric and exhibited a marked low temperature shoulder. Such effect has been reported in the literature and can be explained by strong charge-charge repulsion between the head groups [39]. Contrarily to what was observed with DMPC, DMPG showed a strong perturbation of both the pre-transition and main phase transition by penetratin. The increase in peptide concentration leads to a gradual decrease in the main transition enthalpy to an almost abolishment at P/L ratio of 1/10. A great effect in the Tm was also observed with almost 9°C shift, indicating a strong rigidification of the membrane (Table 2).

In the case of DMPE only the main phase transition can be observed, this transition corresponds to the gel to fluid phase transition. Penetratin induced close to 50% reduction in ΔH and an increase of 3.8°C in Tm (Table 2). As mentioned above, the increase in Tm indicates that penetratin favours the gel *versus* the fluid phase, so it contributes to rigidify the membrane. As for the enthalpy (ΔH) of the transitions, a considerable decrease was observed upon penetratin interaction with DMPG and DMPE (which effect increased with peptide concentration), stronger in the first case, and not much effect was observed in the case of DMPC. The decrease in enthalpy, which was accompanied by an increase in the spectra half-width (data not shown) indicates a decrease in the phase transition cooperativity due to peptide perturbation (intercalation) of the fatty acid chain packing.

### Consequences of peptide binding on lipid ester bond hydration

**Figure 3:**
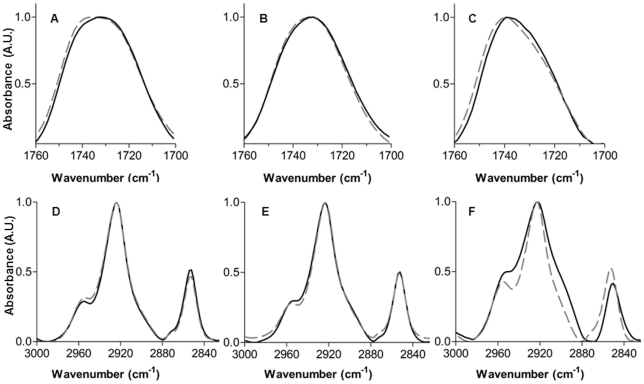
Penetratin effect on phospholipids C = O and C-H vibrations. LUVs spectra in the absence (black line) or presence of penetratin (grey dashed line): Infrared spectra of lipids in the region of C = O stretching vibration. A) PG; B) PC; C) PE. Infrared spectra of lipids in the region of C-H stretching vibration. D) PG; E) PC; F) PE.

Peptide-induced modifications in bilayer hydration were recorded by measuring ester bonds (C = O) stretching vibrations. This vibration is sensitive to the hydrogen-bonding environment of lipids. For PG (Fig. 3A), a single broad carbonyl peak centred around 1733 cm^−1^ was observed. This broad carbonyl peak is composed of two separate components, as indicated by second derivative minima (not shown): a “dehydrated” carbonyl (1743 cm^−1^) and a “hydrated” carbonyl (1724 cm^−1^) [40]. Penetratin induced a shift of the absorption band towards higher wavenumbers (Fig. 3A). Analysis of the second derivative minima indicated that the shift in the absorption band was due to an increase in the proportion of non hydrated carbonyl absorption. The same phenomenon, i.e. an increase in the dehydrated carbonyl absorption, although less intense, was observed with PC LUVs (Fig. 3B). In the case of PE LUVs, the contours of the ester carbonyl band near 1735 cm^−1^ are fairly broad. This band is composed of several components with maxima near 1742, 1722, and 1714 cm^−1^ (Fig. 3C). Upon penetratin interaction, there is a marked decrease in the relative spectral intensity in the low wavenumber range of the band contour, and an increase of the high wavenumber component around 1742 cm^−1^ (Fig. 3C). These data indicates that with the three different phospholipids, penetratin interaction results in a decrease in the C = O hydrogen bonding as a consequence of peptide adsorption to the membranes [40]–[42].

### Ordering effect of penetratin on lipid acyl chains

The C-H stretching vibration of the lipid acyl chains give rise to bands in the spectral region 3100–2800 cm^−1^. The strongest bands correspond to the asymmetric and symmetric CH_2_ stretching at around 2920 and 2850 cm^−1^ [43]. The position of these vibration frequencies illustrates the degree of ordering of the acyl chains in the bilayer. No significant spectral shifts were recorded after penetratin binding to PG or PC LUVs (Fig. 3D,E). Penetratin binding to PE vesicles induced a change in the profile of the C-H stretching region of this lipid. A shift of symmetric and asymmetric CH_2_ bands from 2852 to 2850 cm^-1^ and from 2923 to 2921 cm^−1^ was observed (Fig. 3F). This indicates that the fluidity of the membrane decreases [40], [42], [44].

### Peptide structure in the absence or presence of lipids

As described above, penetratin interacts with PC, PG and PE membranes but, as the effect on the membranes depends on phospholipids composition, the binding mechanism seems to be different. Penetratin is known to be a “structural chameleon” that can modulate its secondary structure according to its environment [31], [33]. Moreover, the structural plasticity of penetratin seems to be important for membrane interaction and internalization processes. Therefore, by means of circular dichroism (CD) and FTIR spectroscopy, we investigated whether the different binding mechanism of penetratin to each type of lipids can be associated with a particular secondary structure.

**Figure 4:**
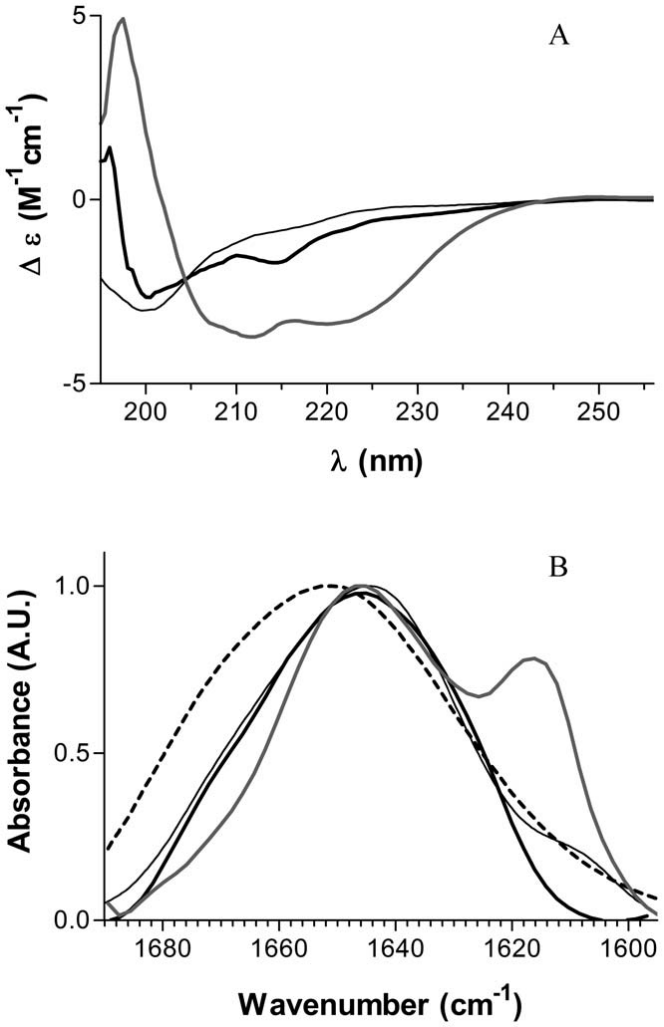
Penetratin structure in the absence or presence of membranes. A. Circular dichroism spectra of penetratin and B. FTIR spectra of penetratin in the absence and presence of LUVs. Peptide in solution in 0.5 mM HEPES buffer, pH 7.4, (black thin line) in the presence of PG LUVs (gray line), PC LUVs (black thick line) and PE LUVs (dashed line).

CD spectrum of penetratin in buffer solution has characteristics of a peptide adopting mainly a random coil structure (Fig. 4A). In the presence of PG LUVs, penetratin CD spectrum shows a transition to an α-helical structure as indicated by two negative peaks at 208 and 222 nm. A negative peak was also observed around 212 nm, which can be attributed to some β-sheet content. In the presence of PC LUVs, penetratin adopted mainly a random coil structure (Fig. 4A) with a negative peak around 214 nm that can be attributed to a β-sheet contribution. Because of the strong aggregation of PE LUVs in the presence of penetratin and problems due to light scattering, we were not able to obtain the CD spectrum. We used instead FTIR spectroscopy to gain information about structural changes of the peptide in the presence of lipids. In the absence of lipids, the infrared spectrum of the peptide in the amide I region was centred at 1644 cm^−1^ (Fig. 4B), consistent with a predominantly random conformation [45]–[47]. A shoulder was observed at 1608 cm^−1^ which could correspond to association of β-sheet structures, favoured by the high concentration of peptide used. In the presence of PG LUVs, the strong absorption band observed at 1616 cm^−1^ and the corresponding shoulder at 1681 cm^−1^ confirmed the presence of intermolecular antiparallel β-sheet structures (Fig. 4B) [45], [48]. The main absorption band with a maximum at 1647 cm^−1^, presented shoulders around 1652 cm^−1^, corresponding to α-helix contribution, as established by CD, and 1637 cm^−1^ and 1672 cm^−1^ indicating the presence of intramolecular antiparallel β-sheet structures. For the peptide bound to PE LUVs, the main absorption band was centred at 1651 cm^−1^ which indicated that penetratin adopted mainly an α-helical structure (Fig. 4B). In the presence of PC LUVs the peptide adopted mainly a random conformation; however, shoulders became visible at 1654 cm^−1^ and 1630 cm^−1^ corresponding to a certain amount of α-helix and β-sheet structures (Fig. 4B).

## Discussion

In this study we characterized the membrane aggregation capacity of penetratin using LUVs of three different compositions: PC and PE main phospholipids of, respectively, the outer and inner leaflets of eukaryotic cells and PG, a phospholipid highly represented in prokaryotes' outer leaflet.

Membrane aggregation experiments by turbidimetry and cytometry showed that penetratin is able to aggregate very efficiently PE and PC but is unable to aggregate PG membranes. This finding contrasts with the report describing the penetratin induced membrane aggregation with DMPG and DOPG LUVs [24]. However, to observe LUVs aggregation, the P/L ratios used by this authors were equal or higher than 1/13.6. The differences in vesicle aggregation were not due to the affinity of the peptide for the membranes because first, the affinity for PE and PG was higher than for PC (which aggregates) and second, the mass changes observed by PWR were very similar for PC and PE membranes and even higher for PG membranes. However, the PWR analysis showed that the binding process for PG was monophasic in contrast to the binding to PE and PC which were biphasic indicating a different interaction mode. This difference correlates with the aggregation capacity and suggests that the peptide-induced lipid perturbation/reorganization and/or peptide secondary structure is different for the three lipids. Therefore, we investigated different structural aspects of these peptide-membrane interactions.

The lipid fluidity studies by Laurdan fluorescence spectral shift (GP) and phase transition temperature shift by DSC revealed that penetratin is able to reduce the lipid fluidity of PE and PG membranes but not (or very little) the fluidity of PC membranes. Penetratin interaction with PC membranes induced only small changes in the transition temperature and fluidity of the membrane, a small change in C = O vibration and no change in C–H stretching. These data is in agreement with the observation of Binder and Lindblom that penetratin does not affect C–H stretching and C = O vibration on DMPC [49]. Overall, the small perturbation of the membrane by the peptide indicates a rather superficial interaction of the peptide in the PC membrane surface without affecting the fatty acid chain region mobility.

Contrarily to what was observed with DMPC, in the case of DMPG, DSC measurements showed a strong perturbation of both the pre-transition and main phase transition temperatures by penetratin which leads to a gradual decrease in the main transition enthalpy with the increase in P/L ratio to an almost abolishment at P/L ratio of 1/10. These data indicates a strong rigidification of the membrane. This was in agreement with the GP changes measured by the peptide induced laurdan shift of the spectra and the strong C = O vibration change. A favourable interaction between the positively charged penetratin amino acids and the negatively charged lipids is evidenced here and is in agreement with many other studies. The rigidification of the membrane by the peptide may be due to the strong electrostatic interaction between the peptide and the lipid head groups that reduces the charge repulsion between the lipid head groups allowing the lipids to become closer. The great effect observed on the main phase transition enthalpy suggests that penetratin affects not only the head group tilting but also the fatty acid chain packing. Penetratin induced a 50% reduction in ΔH and an increase of 3.8°C in Tm for DMPE. As mentioned above, the increase in Tm indicates that penetratin favours the gel *versus* the fluid phase, so it contributes to rigidify the membrane. This data again, correlates with the stronger decrease in membrane fluidity observed with laurdan. The FTIR experiments also showed a higher change in the C = O vibration spectra. It must be noticed that for PE, the C-H stretching was perturbed by the peptide suggesting that it can penetrate deeper into the bilayer compared to the PC and PG. This could arise from the small size of the PE head group, allowing deeper penetration.

Concerning the structural analysis of the peptide in contact with the membranes (CD and FTIR), the results indicate that in contact with PC, penetratin remains quite “unstructured”. In association with PG, both CD and FTIR results show that the peptide adopts both α helix and β sheet structures and that β sheets can interact forming anti parallel structures. This is in agreement with different reports on penetratin showing different degrees of α–helix and β–sheet structures in PG containing membranes [19], [24], [31], [32]. Finally, in the presence of PE the peptide acquires an α-helical conformation. A comparison of the PWR magnitude of the spectral shift observed with *p*- and *s*-polarized light in the second binding event (Table 1) indicates that the shifts obtained with *s*-polarized light are larger than those obtained with *p*-polarized light. Such spectral changes may indicate that the peptide is placed with its long axis parallel to the lipid bilayer.

**Figure 5:**
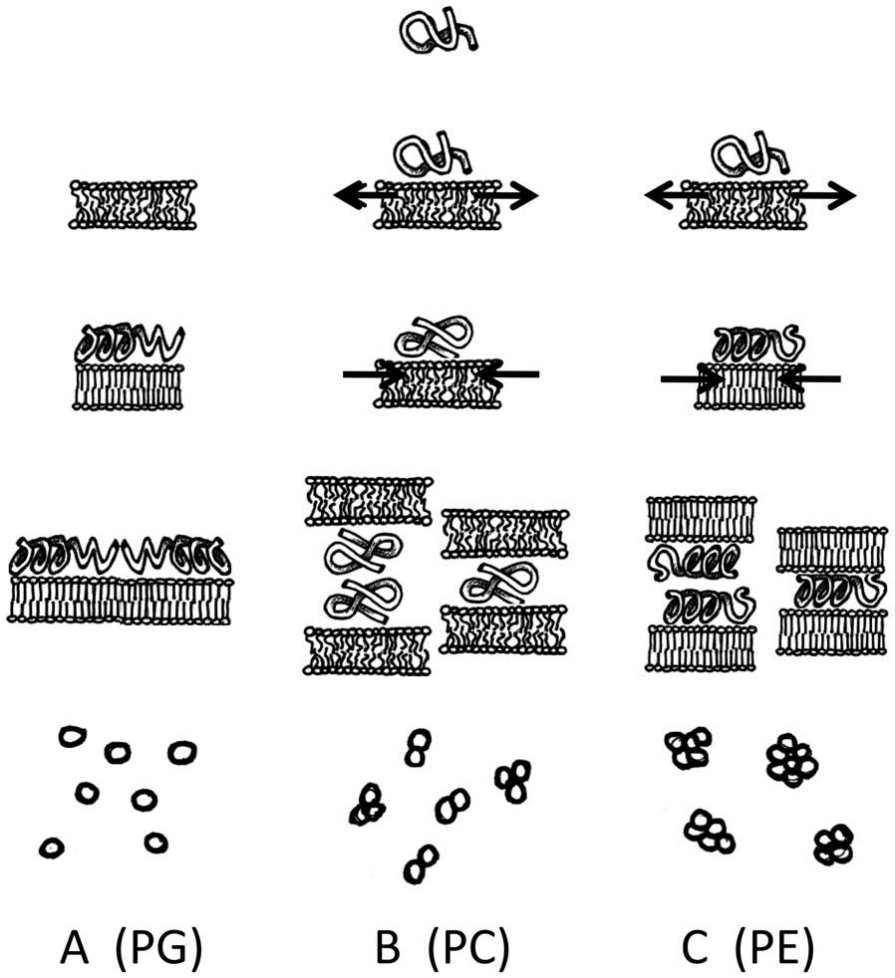
Model for penetratin-phospholipid membranes interactions. A) The association of penetratin with PG membranes results in the conformational change of the peptide with α-helix and β-sheet contributions and a decrease in membrane fluidity. B) With PC membranes, the peptide associates in a two step processes but remains quite unstructured and does not change notably the fluidity of the membrane. It induces membrane aggregation. C) With PE penetratin binds membranes in a two step processes with an accompanying structural change (mainly α-helix). The membrane experiments a decrease in fluidity and strong membrane aggregation is allowed. Arrows indicate the phospholipid movements induced by penetratin as observed by PWR. The increase in membrane rigidity is indicated by the straight lines of lipid acyl chains. The small circles at the bottom represent membrane vesicles and their degree of aggregation induced by penetratin. For more details see the discussion.

Altogether, these data indicate that penetratin is a versatile peptide that is able to induce different changes depending on the nature of the membrane lipids. A putative model of interaction is proposed in figure 5. The relatively unstructured peptide in solution is able to interact with different phospholipids and to adopt different structures. In the case of negatively charged phospholipids (PG), penetratin experiments a one-step binding, mainly by electrostatic interaction. Peptide binding results in a decrease of mobility of the bound phospholipid with the consequent decrease of membrane fluidity. At the same time, the peptide becomes structured with α–helical and β–sheet contributions. This conformation allows antiparallel interaction of peptide molecules on the membrane surface but does not allow interaction with other membranes precluding vesicle aggregation (Fig. 5A). With zwitterionic phospholipids, penetratin will be able to bind by electrostatic and non electrostatic interactions resulting in a two-step binding. In the first step, the peptide will be able to separate the lipids and in the second step the phospholipids redistribute again in a more compact bilayer probably by the structural change of the peptide. However, depending on the nature of the lipid, the peptide will acquire different conformation. In the case of PC, the membrane remains quite fluid and the peptide remains “unstructured” but different than in solution. Notice that the CD and FTIR spectra of penetratin in solution are different in solution and when interacting with PC. This situation allows membrane aggregation induced by one peptide interacting with two membranes or by homotopic peptide dimerization (Fig. 5B). For PE, during the second step, penetratin increases its α–helical structure allowing enough compaction of the membrane and resulting in an important decrease of fluidity. This difference compared to PC, could be due to the smaller head group of the PE. Then, as in the case with PC, membrane aggregation will result from one or two peptide molecules bridging (Fig. 5C).

Considering the presented data, we can speculate on the importance of the different penetratin properties in different situations. For the antimicrobial activity [25], there is not enough data in the literature to allow a mechanistic explanation. However, the presence of β–structures and membrane rigidity observed with PG membranes will serve as basis for future interpretations on the toxic effect of penetratin. For the export of transduction proteins, the capacity of penetratin to bridge membranes could participate in membrane pinching on rich PE membrane domains. There is also little information concerning the export of transduction proteins. For cell penetration, penetratin interacts with a PC rich membrane and three different points merit discussion. First, the data indicates that the structuration on α or β structures is not important for penetration. This is in agreement with the study comparing Tat, R7W and penetratin that shows a negative correlation between α–helicity and efficient peptide internalization [29], and with the study showing that a coil structure will be related to direct translocation [50]. Second, the membrane decrease in fluidity may not be important. Moreover, a strong rigidification could block local membrane deformations necessary for peptide internalization. Additionally, membrane rigidification in certain membrane domains could lead to lateral membrane heterogeneity with regions of low tension in between different domains that the peptide may use to more easily perturb the membrane as suggested by different studies [51]–[53]. Third, the capacity of the peptide to induce membrane bridging (i.e. structures in which the peptide is covered with phospholipids), could be very important to provoke the membrane deformations such as membrane curvature, tubulation and inverted micelles formation necessary for cellular uptake.

## Materials and Methods

### Materials

Dimyristoylphosphatidylcholine (DMPC), dimyristoylphosphatidylglycerol (DMPG) and dimyristoylphosphatidylethanolamine (DMPE) were purchased from Genzyme (Switzerland). Dioleoylphosphatidylethanolamine (DOPE) was purchased from Avanti Lipids (Alabama, USA). L-α-phosphatidylcholine (PC), L-α-phosphatidyl-DL-glycerol (PG) and L-α-phosphatidylethanolamine (PE) from egg yolk, and deuterium oxide were purchased from Sigma. Laurdan was purchased from Molecular probes. Penetratin (RQIKIWFQNRRMKWKK) was synthesized using Boc solid phase strategy and was purified by HPLC as previously described [20].

### Preparation of membranes

Multilamellar vesicles (MLVs) were obtained by dissolving the appropriate amounts of lipids in a mixture of chloroform and methanol, 2/1 (v/v), followed by solvent evaporation under nitrogen. Final traces of solvent were removed in a vacuum chamber attached to a liquid nitrogen trap for 3–4 h. Lipid films were hydrated with 10 mM HEPES pH 7.4 and vortexed extensively at a temperature above the phase transition temperature of the lipid to obtain MLVs. Large unilamellar vesicles (LUVs) of different composition were prepared by extrusion of MLVs through a polycarbonate filter (pore diameter 100 nm) as described in [54]. Laurdan was added at a lipid molar ratio of 1/100. The peptide was added to LUVs (aggregation, fluorescence or FTIR experiments), planar bilayers (PWR studies) or MLVs after their formation (DSC studies) to obtain the required peptide/lipid molar ratio.

### Differential Scanning Calorimetry

Calorimetry was performed on a high-sensitivity Differential Scanning Calorimeter (Calorimetry Sciences Corporation). A scan rate of 1°C/min was used and there was a delay of 10 min between sequential scans in a series that allows for thermal equilibration. Data analysis was performed with the fitting program CPCALC provided by CSC and plotted with Igor. The total lipid concentration used was 1 mg/ml. For peptide concentrations corresponding to P/L 1∶10, no thermal events were observed over the temperature range of 0–100°C. This indicates that the endothermic events observed in this study arise solely from phase transitions of the phospholipids vesicles. A minimum of three heating and cooling scans were performed.

### Fluorescence spectroscopy

Fluorescence measurements were performed with a Cary fluorimeter (Varian). The excitation and emission band-pass were set at 5 nm. Spectra were recorded 10 min after addition of penetratin to LUVs (P/L molar ratio 1/25), using a 1 cm path length quartz cuvette, thermostated at 37°C or 25°C. All fluorescence spectra were corrected for the baseline signal. Laurdan emission spectra were recorded from 400 to 600 nm using a 365 nm excitation wavelength in the absence or presence of penetratin in 0.5 mM HEPES buffer (pH 7.4). The excitation generalized polarization (GP) was calculated as
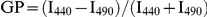



where I_440_ and I_490_ are the fluorescence intensities at the maximum emission wavelength in the ordered (440 nm) and disordered (490 nm) phases [38].

### Fourier transformed infrared spectroscopy

LUVs were prepared as described above, using 0.5 mM HEPES-^2^H_2_O (p^2^H 7.4) buffer. The p^2^H was measured with a glass electrode and was corrected by a value of 0.4 according to [55]. The liposome suspension was mixed with penetratin at a P/L molar ratio of 1/30 and incubated at 30°C for 10 min. For control experiments, spectra of the liposome suspension or of the peptide dissolved in 0.5 mM HEPES-^2^H_2_O (p^2^H 7.4) buffer were also recorded. To avoid spectral contribution of unbound peptide, removal of unadsorbed peptide was performed by centrifugation at 160 000×*g* for 40 min (Beckman Airfuge). The pellet was resuspended in 24 µl of HEPES-^2^H_2_O buffer.

Samples were loaded between two CaF_2_ circular cells, with a 50 µm Teflon spacer. FTIR spectra were recorded with a Nicolet 510 M FTIR spectrometer which was continuously purged with dry air. The nominal spectral resolution was 4 cm^−1^; 256 scans were collected and co-added per sample spectrum, and Fourier-transformed for each sample. Every infrared spectrum was representative of at least three independent measurements. The infrared spectra of the corresponding buffer and residual water vapour were subtracted from the infrared spectrum of the sample. Peak position was determined using second derivative minima.

### Circular dichroism spectroscopy

CD spectra were recorded with a Jobin Yvon CD6 dichrograph. The instrument outputs were calibrated with D(+)−10-camphorsulfonic acid. The samples were scanned in a quartz optical cell with a 1 mm path length and recorded from 195 to 260 nm with 0.5 nm step. The measurements were performed at 37°C. Four scans were accumulated and averaged after buffer (or LUVs) spectra subtraction and baseline correction. Each presented spectrum is the average of 3 independent measurements. The CD spectra were recorded in 0.5 mM HEPES buffer (pH 7.4), at a peptide concentration of 43 µM and a peptide/lipid molar ratio of 1/25. CD measurements are reported as delta ε (M^−1^ cm^−1^) per residue.

### Plasmon waveguide resonance (PWR) spectroscopy

PWR spectra are produced by resonance excitation of conduction electron oscillations (plasmons) by light from a polarized CW laser (He-Ne; wavelength of 632.8 and 543.5 nm) incident on the back surface of a thin metal film (Ag) deposited on a glass prism and coated with a layer of SiO_2_ [56]. Experiments were performed on a beta PWR instrument from Proterion Corp. (Piscataway, NJ) that had a spectral resolution of 1 mdeg. The sample to be analyzed (a lipid bilayer membrane) was immobilized on the resonator surface and placed in contact with an aqueous medium, into which penetratin was introduced. The self assembled lipid bilayers were formed as previously described [35]. PWR spectra, corresponding to plots of reflected light intensity versus incident angle, can be excited with light whose electric vector is either parallel (s-polarization) or perpendicular (p-polarization) to the plane of the resonator surface. Spectral simulation [56] and/or graphical analysis [57] allow one to obtain information about changes in the mass density, structural asymmetry, and molecular orientation induced by bimolecular interactions occurring at the resonator surface. Here, the graphical analysis method was employed [35].

Affinities between the peptide and the lipids were obtained by plotting the PWR spectral changes that occur upon incremental additions of ligand to the cell. Since the PWR is only sensitive to the optical properties of material that is deposited on the resonator surface, there is no interference from the material that is in the bulk solution. Data fitting (GraphPad Prism) through a hyperbolic saturation curve provides the dissociation constants. It should be noted that since concomitantly with the binding process other processes, such as membrane reorganization and solvation occur the dissociation constants correspond to apparent dissociation constants.

### LUVs aggregation measurements

LUVs aggregation was monitored by turbidimetry (absorbance at 340 nm) with a Cary spectrophotometer (Varian) as described [58]. Different quantities of penetratin were added to a 100 µl quartz cuvette containing 2 µg lipids in a HEPES 0.5 mM pH 7.4 buffer to obtain the desired peptide/lipid ratios and the absorbance was followed until it reached a plateau (30 minutes after peptide addition).

A second method was used to measure the aggregation of LUVs induced by penetratin. 2 µg of penetratin were incubated with 10 µg of LUVs in 500 µl of buffer (0.5 mM HEPES buffer, pH 7.4). After 20 min of incubation at room temperature the samples were analyzed by flow cytometry as previously described [59]. Briefly, the analysis of the forward scatter (FSC) and the side scatter (SSC) was performed using a LSR II cytometer (Beckton Dickinson) equipped with a 15 mW 488 nm air cooled argon ion laser. A constant SSC detector was used. FSC was set in log scale. For each experiment, 5 000 events were collected.
